# Slug-Dependent Upregulation of L1CAM Is Responsible for the Increased Invasion Potential of Pancreatic Cancer Cells following Long-Term 5-FU Treatment

**DOI:** 10.1371/journal.pone.0123684

**Published:** 2015-04-10

**Authors:** Kaja Lund, Jennifer L. Dembinski, Nina Solberg, Alfonso Urbanucci, Ian G. Mills, Stefan Krauss

**Affiliations:** 1 Unit for Cell Signaling, Institute of Microbiology, Cancer Stem Cell, Innovation Center (CAST), Rikshospitalet, Oslo, Norway; 2 Centre for Molecular Medicine Norway, Nordic European Molecular Biology Laboratory Partnership, Forskningsparken, University of Oslo, Oslo, Norway; 3 Department of Cancer Prevention, Institute for Cancer Research, Oslo University Hospital, Oslo, Norway; 4 Department of Urology, Oslo University Hospital, Oslo, Norway, Centre for Molecular Medicine of Urology, Oslo University Hospital, Oslo, Norway; 5 Norwegian Institute of Public Health, Division of Infectious Disease Control, Department of Bacteriology and Immunology, Oslo, Norway; University of Alabama at Birmingham, UNITED STATES

## Abstract

**Background:**

Pancreatic adenocarcinoma is a lethal disease with 5-year survival of less than 5%. 5-fluorouracil (5-FU) is a principal first-line therapy, but treatment only extends survival modestly and is seldom curative. Drug resistance and disease recurrence is typical and there is a pressing need to overcome this. To investigate acquired 5-FU resistance in pancreatic adenocarcinoma, we established chemoresistant monoclonal cell lines from the Panc 03.27 cell line by long-term exposure to increasing doses of 5-FU.

**Results:**

5-FU-resistant cell lines exhibited increased expression of markers associated with multidrug resistance explaining their reduced sensitivity to 5-FU. In addition, 5-FU-resistant cell lines showed alterations typical for an epithelial-to-mesenchymal transition (EMT), including upregulation of mesenchymal markers and increased invasiveness. Microarray analysis revealed the L1CAM pathway as one of the most upregulated pathways in the chemoresistant clones, and a significant upregulation of L1CAM was seen on the RNA and protein level. In pancreatic cancer, expression of L1CAM is associated with a chemoresistant and migratory phenotype. Using esiRNA targeting L1CAM, or by blocking the extracellular part of L1CAM with antibodies, we show that the increased invasiveness observed in the chemoresistant cells functionally depends on L1CAM. Using esiRNA targeting β-catenin and/or Slug, we demonstrate that in the chemoresistant cell lines, L1CAM expression depends on Slug rather than β-catenin.

**Conclusion:**

Our findings establish Slug-induced L1CAM expression as a mediator of a chemoresistant and migratory phenotype in pancreatic adenocarcinoma cells.

## Introduction

Pancreatic adenocarcinoma is an extremely deadly disease. The early course of the disease is often asymptomatic leading to only 8% of cases being diagnosed at this stage. The outlook for late-stage adenocarcinoma patients is bleak, with only 20% of patients being candidates for surgery (due to late diagnosis/tumor metastasis), resulting in a 5-year survival of less than 5% [1]. Current treatment options available may extend survival and relieve symptoms in patients, but are not curative in most cases.

5-Fluorouracil (5-FU) has for a long time been an established form of chemotherapy for pancreatic adenocarcinoma, together with the drug gemcitabine [2]. However, inherent (de novo) and acquired resistance are major obstacles for the success of 5-FU based chemotherapy in pancreas adenocarcinoma and other tumors [3]. Acquired drug resistance, which develops during treatment, is often manifested by several resistant mechanism and is therefore therapeutically difficult to reverse.

5-FU decreases the biosynthesis of pyrimidine nucleotides by inhibiting thymidylate synthase (TS), an enzyme that catalyzes the rate-limiting step in DNA synthesis [4]. Although the mechanisms of resistance to 5-FU remains unclear, several reports have linked chemoresistance in various solid tumor cell lines to epithelial-to-mesenchymal transition (EMT) [5–8]. EMT is a fundamental embryological process characterized by alterations in morphology, cellular architecture, signaling and adhesion leading to a migratory phenotype [9]. When EMT occurs in tumor cells, these cells lose their epithelial features and acquire a more invasive and migratory phenotype leading to augmented metastatic potential. Molecular markers for EMT include increased expression of vimentin and N-cadherin and increased expression of transcription factors that repress E-cadherin expression, including Twist, Snail, and Slug [10].

The L1 cell adhesion molecule (L1CAM) is a highly conserved transmembrane glycoprotein of the immunoglobulin superfamily that was first identified to play a part in the development and regeneration of neuronal tissue [11]. L1CAM expression has been observed in a number of cancer cell lines and tissues, and high L1CAM expression is often associated with poor prognosis and short survival times [12]. L1CAM has been linked to EMT in several different cancer types, including pancreatic cancer [13–18]. In particular, L1CAM has been associated with a chemoresistant and migratory phenotype in pancreatic ductal adenocarcinoma (PDAC) [19–21]. To investigate the mechanisms involved in the acquisition of 5-FU resistance, we established 5-FU-resistant clones from the pancreatic adenocarcinoma cell line Panc 03.27, and subjected the cell lines to functional tests and microarray analysis. The chemoresistant Panc 03.27 cells underwent phenotypic changes consistent with an EMT, and the expression of EMT-related markers, particularly L1CAM, increased substantially. Knockdown studies showed that the L1CAM expression in the 5-FU-resistant clones was dependent on the transcription factor Slug but not on β-catenin, and knockdown of L1CAM confirmed a functional link between L1CAM and the proliferative and invasive potential of the chemoresistant Panc 03.27 clones. Knockdown studies further showed that L1CAM moderately protected chemoresistant B1V cells from apoptosis induced by 5-FU. Our findings provide further insight into the molecular mechanisms leading to a chemoresistant and migratory phenotype in pancreatic cancer cells and highlight the importance of addressing Slug-induced L1CAM expression in recurrent PDAC.

## Results

### Development of 5-FU-resistant clones

**Fig 1:**
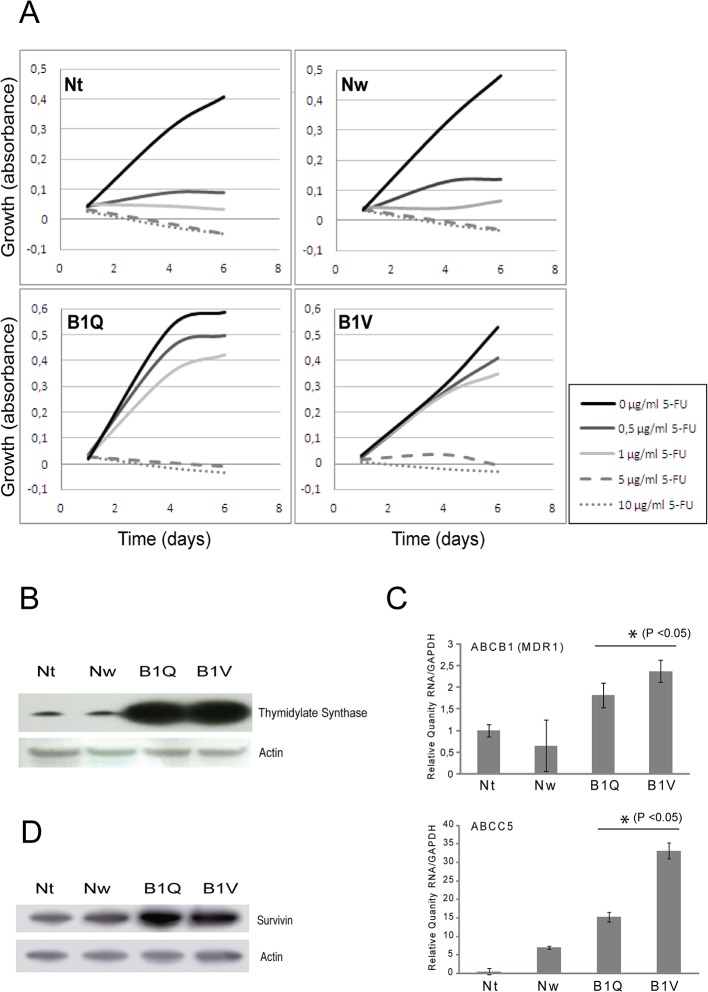
5-FU-resistant clones display resistance mechanisms. (A) The chemosensitive (Nt, Nw) and resistant (B1Q and B1V) cell lines were treated with 0–10 μg/ml 5-FU) over 6 days. Graph displays growth vs. days of exposure. (B) Western blot showing the levels of Thymidylate Synthase (TS) in the chemosensitive and the chemoresistant cell lines. (C) RNA levels (relative quantity) of ABCB1 (MDR1) and ABCC5 as measured by RT-PCR. (D) Western blot showing the levels of survivin in the chemosensitive and the chemoresistant cell lines. Error bars represent standard deviation. Statistically significant difference between the chemosensitive and the chemoresistant clones (P <0.05) is indicated by *.

Panc 03.27 5-FU-resistant cell lines were generated by continuous exposure of the tumor cells to 5-FU over a 6 month period, starting at 0.5 μg/ml 5-FU and increased to 1 μg/ml over time. To verify the dose dependent chemoresistance of the obtained clones, a dose curve was run to a range of 5-FU concentrations (0.5–10 μg/ml) over a 6 day period. As expected, clones that were grown without 5-FU selection (named Nt and Nw) were sensitive to all concentrations of 5-FU. In the chemoresistant clones B1Q and B1V normal growth was seen with doses up to 1 μg/ml 5-FU, while the higher concentrations of 5-FU (5–10 μg/ml) still inhibited growth *(Fig 1A)*.

### 5-FU-resistant clones display resistance mechanisms

5-FU sensitivity has been shown to be inversely related to the level of thymidine synthase (TS) protein in cancer cells, and 5-FU-resistant tumors commonly express high levels of TS protein [22,23]. High expression of TS in cancer tissue is an indicator of poor prognosis for 5-FU-based chemotherapy for colorectal cancer patients [24]. The protein level of TS was analyzed in all four clones. TS was drastically increased in the chemoresistant clones B1Q and B1V, as shown by Western blot analysis *(Fig 1B)*. Using taqman RT-PCR, we next analyzed the RNA levels of efflux pumps associated with 5-FU- and multidrug resistance [25,26]. We observed a statistically relevant increase in the expression of the efflux pumps ABCB1 (MDR1) and ABCC5 (MRP5) in the chemoresistant clones as compared to the chemosensitive clones *(Fig 1C).* The level of survivin was also greatly increased in the chemoresistant clones as assessed by Western blotting *(Fig 1D).* Survivin is an inhibitor of apoptosis expressed in the G2/M phase of the cell cycle and overexpression of survivin is linked to resistance to apoptotic stimuli induced by chemotherapeutic drugs [27,28].

### 5-FU-resistant clones display morphological changes that are associated with EMT

**Fig 2:**
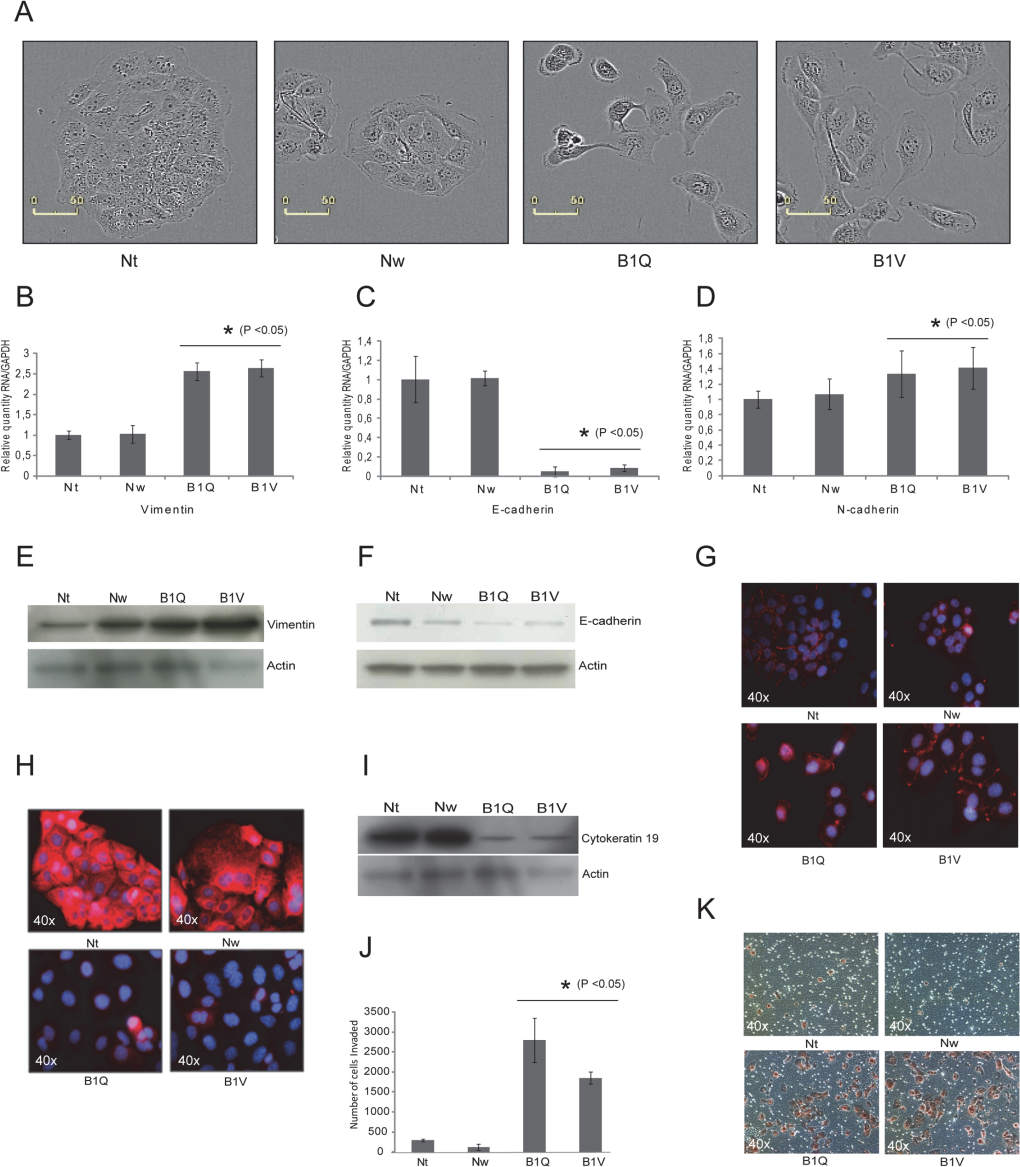
5-FU-resistant clones display morphological changes that are associated with EMT. (A) Phase contrast photos showing cell morphology of normal (Nw, Nt) and chemoresistant clones (B1Q, B1V), 40x. (B-D) RNA levels (relative quantity) of vimentin, E-cadherin, and N-cadherin, as measured by RT-PCR. Error bars represent standard deviation. Statistically significant difference between the chemosensitive and the chemoresistant clones (P <0.05) is indicated by *. (E-G) Western blot and immunostain showing the levels of vimentin, E-cadherin, and N-cadherin, in the chemosensitive (Nt, Nw) and the chemoresistant (B1Q, B1V) cell lines. (H) Immunostain and (I) Western blot showing levels of CD19 in the chemosensitive and the chemoresistant cell lines. (J) Graph displaying the results of 24 hour invasion assays performed on the clones, with phase contrast images (20x) showing representative areas of invading cells stained with crystal violet stain (K). Assay was performed three times. Statistically significant difference between the chemosensitive and the chemoresistant clones (P <0.05) is indicated by *.

When the morphology of the 5-FU-resistant clones was compared to the sensitive clones, the former displayed a more mesenchymal-like phenotype, with cell scattering and increased formation of pseudopodia, while the sensitive clones display a tightly packed epithelial morphology *(Fig 2A)*. The 5-FU-resistant cells appeared larger and more stretched out than the sensitive cells. The mesenchymal-like phenotype, which was maintained for over 30 passages, is in line with previous observations of drug-induced chemoresistance in cell lines [29,30].

Important hallmarks of EMT are a downregulation of the epithelial marker E-cadherin accompanied by an upregulation of N-cadherin and vimentin [9]. Accordingly, the chemoresistant clones showed an increase in vimentin *(Fig 2B and 2E)* and N-cadherin *(Fig 2D and 2G)* on both protein and RNA levels, and a downregulation of E-cadherin *(Fig 2C and 2F).* Cytokeratin 19 (CK19) is an intermediate filament that is in part responsible for the structural integrity of cells. Various cytoskeletal modifications within a cell are associated with malignant transformation, and a loss of CK19 has been associated with EMT [31] and multidrug resistance [32]. When the Nt, Nw, B1Q and B1V clones were analyzed for the expression of CK19 by immunocytochemistry and Western blot, a massive reduction of CK19 was found in the chemoresistant clones as compared to the 5-FU sensitive clones *(Fig 2H and 2I).* To evaluate if the changed morphology that was observed in the chemoresistant clones was functionally significant, we examined the invasive properties of the clones using a matrigel invasion assay *(Fig 2J and 2K).* Chemoresistant clones B1Q and B1V were 4 to 6 times more invasive than the two chemosensitive clones Nt and Nw.

### L1CAM is upregulated in the 5-FU-resistant cells

**Fig 3:**
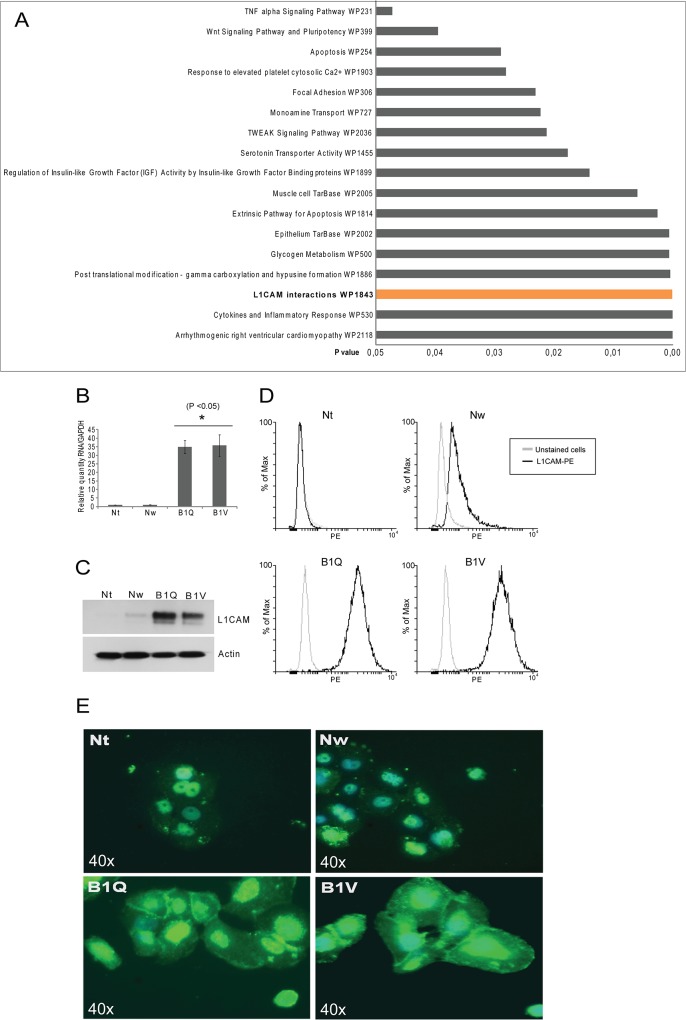
L1CAM is upregulated in the 5-FU-resistant cells. (A) Gene Ontology analysis of 319 genes upregulated in the B1V clone compared to the Nt clone of the Panc 03.27 cell line. (B) RNA levels (relative quantity) of L1CAM in the chemosensitive (Nt, Nw) and the chemoresistant (B1Q, B1V) cell lines, as measured by RT-PCR. Error bars represent standard deviation. Statistically significant difference between the chemosensitive and the chemoresistant clones (P <0.05) is indicated by *. (C)Western blot showing levels of L1CAM in all cell lines. (D) Flow cytometric analysis using anti L1CAM-PE antibody on all cell lines. Unstained cells are shown in grey and antibody-stained cells are shown in black. (E) Immunostain of L1CAM. Increased membrane localization of L1CAM can be seen in the of the chemoresistant lines (B1Q, B1V). Images are taken with 40x magnification.

In order to establish a more exhaustive molecular profile between the sensitive and non-sensitive cell lines, we performed microarray analysis including the 5-FU resistant cell line B1V and the chemosensitive clone Nt. 607 genes were considered as they presented with log2 change > 1 *(S1 Fig)*. 319 genes were found to be upregulated and 288 genes were downregulated at least 2-fold in B1V compared to Nt (P <0.05) *(S1 and S2 Tables, respectively)*. Gene Ontology analysis of the 319 upregulated genes revealed that the L1CAM interaction pathway was one of the most upregulated pathways in the chemosresistant clones, together with cytokine and inflammatory responses *(Fig 3A)*. L1CAM itself was upregulated 4 times in the B1V cells compared to the Nt cells. Hallmarks of Wnt signaling and pluripotency, apoptosis, serotonin transporter activity, monoamine transport and glycogen metabolism were also found to be upregulated in the B1V chemoresistant cell line *(Fig 3A and S1 Table).*

The increase in L1CAM in the chemoresistant clones was confirmed by real-time PCR (P<0.05) and Western blot analysis (*Fig 3B and 3C, respectively)*. Furthermore, flow cytometric analysis using a PE-conjugated anti-L1CAM antibody showed enrichment of L1CAM positive cells in the B1Q and B1V cell lines *(Fig 3D)*. In support of this observation, immunohistochemistry revealed a distinct L1CAM expression in the membrane of the chemoresistant clones B1Q and B1V but not in the chemosensitive cells Nt and Nw *(Fig 3E).* A proportion of the chemoresistant cells also exhibited nuclear L1CAM immunoreactivity.

### L1CAM is involved in proliferation and invasiveness of the 5-FU-resistant clones, and moderately protects the chemoresistant cells from 5-FU-induced apoptosis

**Fig 4:**
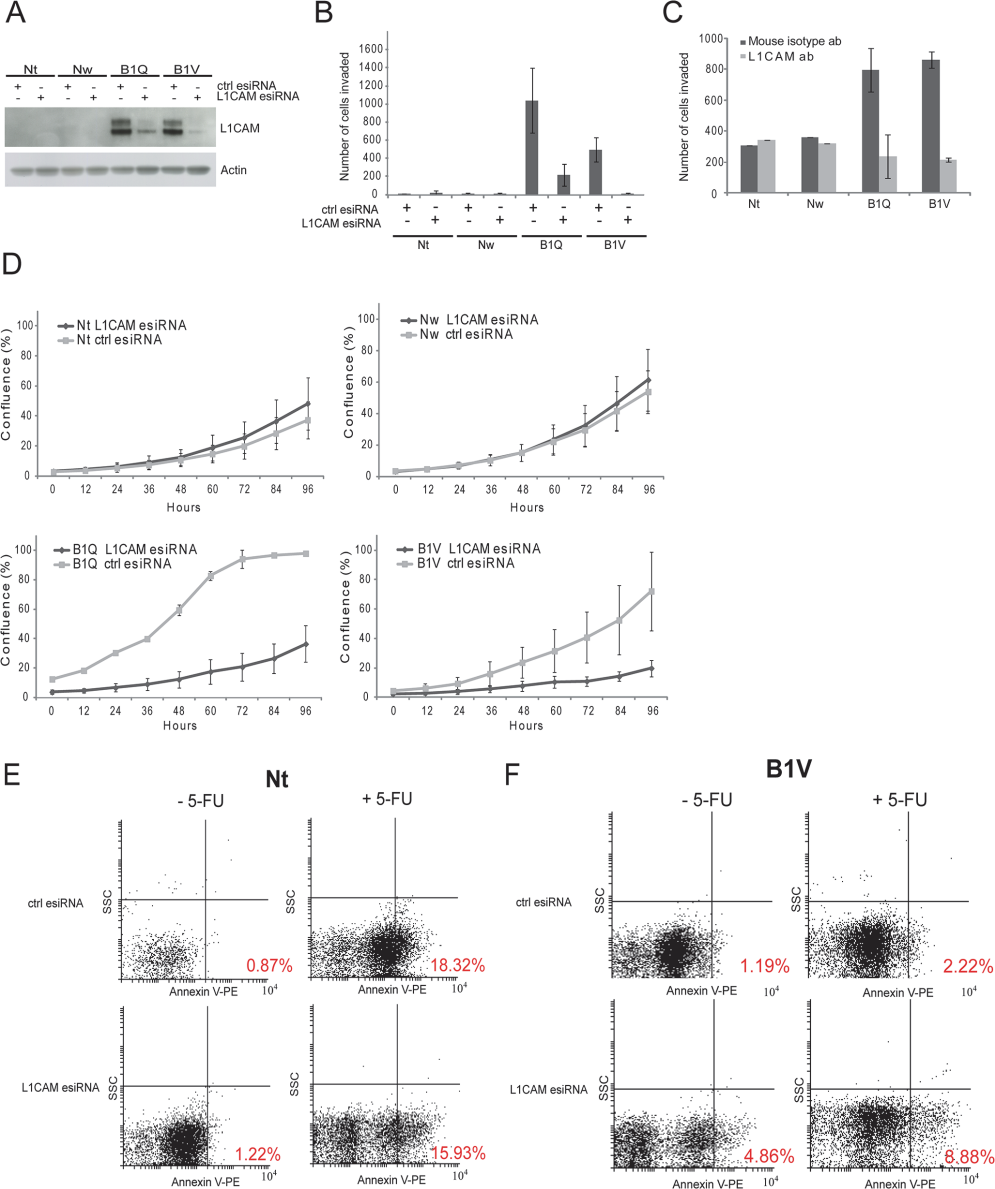
L1CAM is involved in proliferation and invasiveness of the 5-FU-resistant clones. (A) Control western blot showing the levels of L1CAM following transfection with esiRNA targeting L1CAM or control esiRNA, as described in *Materials and methods*. Actin is used as loading control. (B) Graph displaying number of invading cells (24 h invasion assays) following transfection with esiRNA targeting L1CAM or control esiRNA. Experiments were performed twice with similar results. Representative results are shown. (C) Graph displaying numbers of invading cells (48 h invasion assays) of cells treated with 2 μg/ml anti-L1CAM antibody (clone UJ127.11) or control isotype antibody for 1 h at room temperature before they were added to the invasion assay plate. (D) Incucyte growth curves (96 hours) of all cell lines following transfection with esiRNA targeting L1CAM or control esiRNA. The experiment was performed in triplicates. (E) The chemosensitive cell line Nt and the chemoresistant cell line B1V (F) were treated with 1 ug/ml 5-FU for 72 h following transfection with esiRNA targeting L1CAM or control, and Annexin V flow cytometric assays were performed. Each assay was repeated twice with similar results. Percentage in the graphs shows Annexin V-positive cells.

EMT has been associated with an increased invasiveness of tumor cells [33], and previously published reports showed that L1CAM triggers cell migration and invasion in several tumor types [15,18,34]. To investigate whether the upregulation of L1CAM is involved in the increased invasion potential of the chemoresistant cells, the expression of L1CAM was knocked down using esiRNA. esiRNA are endoribonuclease-prepared siRNA pools comprised of a heterogeneous mixture of siRNAs that all target the same mRNA sequence. esiRNA treatment dramatically reduced the protein level of L1CAM in the chemoresistant cell lines B1Q and B1V *(Fig 4A)*, compared to cells transfected with control esiRNA (targeting EGFP). When assayed for invasiveness in a matrigel invasion chamber the number of invasive cells after L1CAM knockdown was significantly lower compared to the negative control after 24 h invasion *(Fig 4B).* To further assess the relationship between the invasiveness of the chemoresistant clones and L1CAM expression, cells were treated with either an antibody targeting the extracellular part of L1CAM (clone UJ127.11), or a mouse isotype control antibody, prior to an invasion assay experiment (invasion assay times were increased to 48 hours). Our results show that treating B1Q and B1V cells with the L1CAM antibody significantly reduced their invasiveness compared to treatment with the control antibody. By contrast, treating chemosensitive Nt and Nw cells with L1CAM antibody had no effect on invasiveness *(Fig 4C).*

Overexpression of L1CAM has been shown to promote tumor cell proliferation, and accordingly, inhibition of L1CAM expression or function can suppress proliferation [35–37]. To investigate whether knockdown of L1CAM had an influence on cell growth in the chemoresistant cells, we performed *in vitro* proliferation assays. All cell lines were subjected to L1CAM and control esiRNA treatment, and the percentage confluence was measured over time by an IncuCyte automated reader *(Fig 4D)*. Measurements started 24 hours after esiRNA transfection. We have previously observed that the effect of esiRNA knockdown on protein level gradually diminishes after 72 hours (results not shown), but to obtain a proper growth curve the experiments were run for at least 96 hours post transfection. The growth curves showed no effect of the esiRNA treatment targeting L1CAM on the chemosensitive cell lines Nt and Nw. However, the chemoresistant cell lines B1Q and B1V displayed significantly reduced growth following L1CAM knockdown *(Fig 4D)* demonstrating that L1CAM expression is involved in cell proliferation of the chemoresistant cells.

When the anti-L1CAM antibody that inhibited invasion in the chemoresistant cells was added to *in vitro* proliferation assays, we could not see any difference in percentage cell confluence between cells treated with the antibody compared to cells treated with the control antibody. This was performed on all four cell lines.

L1CAM has been shown to play a role in the mediation of chemoresistance against gemcitabine and etoposide in PDAC cell lines [38]. To assess whether the L1CAM was involved in mediating resistance to 5-FU in the chemoresistant Panc 03.27 cell lines, Annexin V flow cytometric assays were carried out in Nt and B1V cells 72 hours after cells were treated with L1CAM or control esiRNA in the presence and absence of 1 μg/ml 5-FU. As expected, Nt cells treated with a control esiRNA showed a significant shift towards Annexin V positive cells after being exposed to 5-FU (from 0.87% to 18.32%). In contrast, control treated B1V cells did only show a slight increase (from 1.19% to 2.22%) in Annexin V positive cells in response to 5-FU treatment *(Fig 4E and 4F)*. Knockdown of L1CAM did not significantly alter the rates of apoptosis in the chemosensitive cell line Nt, either in the presence or absence of 5-FU treatment when compared to the ctrl knockdown *(Fig 4E)*. However, knockdown of L1CAM increased percentage Annexin V positive cells, both with or without 5-FU treatment. Thus, L1CAM appears to moderately protect chemoresistant B1V cells from apoptosis in the absence of 5-FU, and more so in the presence of 5-FU *(Fig 4F).*

### Transcriptional regulation of L1CAM depends on Slug, but not on β-catenin

**Fig 5:**
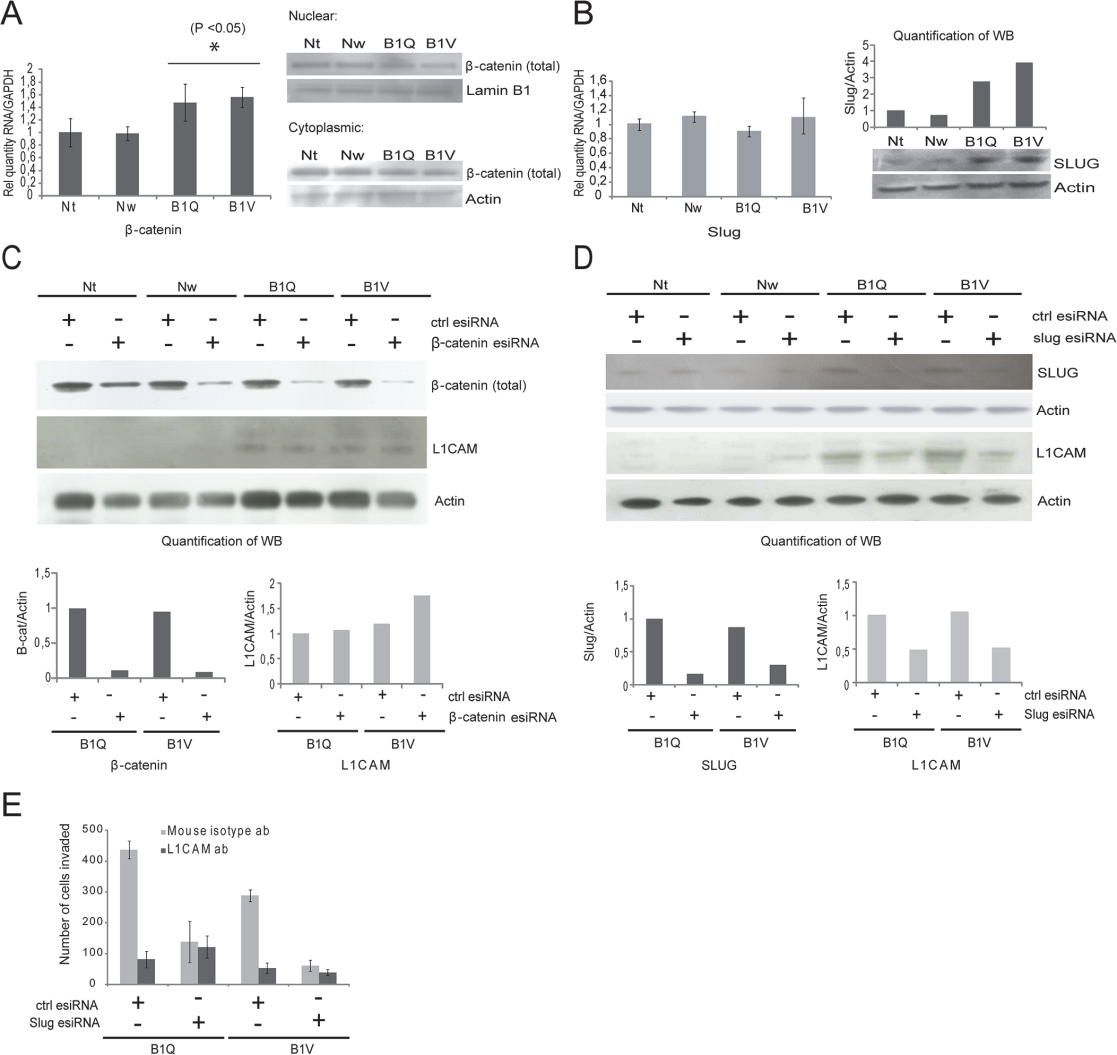
Transcriptional regulation of L1CAM depends on Slug, but not β-catenin. (A) RNA levels (relative quantity, RT-PCR) and western blot showing protein levels of β-catenin in the chemosensitive (Nt, Nw) and the chemoresistant (B1Q, B1V) cell lines. (B) RNA levels (relative quantity, RT-PCR) and western blot showing protein levels of Slug in the chemosensitive (Nt, Nw) and the chemoresistant (B1Q, B1V) cell lines. Protein levels of Slug in the western blot were quantified using Image Studio Lite (version 3.1.4) (normalized against loading controls). (C) Levels of L1CAM following transfection with esiRNA targeting β-catenin or control esiRNA. (D) Levels of L1CAM following transfection with esiRNA targeting Slug or control esiRNA. Quantification of band intensities can be seen in the lower panels in (C) and (D). (E) Graph displaying number of invading cells (24 h invasion assays) following transfection with esiRNA targeting Slug or control esiRNA, in combination with 2 μg/ml anti-L1CAM antibody (UJ127.11) or control isotype antibody for 1 h at room temperature. Experiments were performed twice with similar results. Representative results are shown. Actin is used as loading control in all western blots. Error bars in the RT-PCR graphs represent standard deviation. Statistically significant differences (P <0.05) between the chemosensitive and the chemoresistant clones are indicated by *.

Previous studies have implicated both Slug and β-catenin in the transcriptional regulation of L1CAM [14,39–41]. Slug is a transcriptional factor that is involved in EMT and invasiveness in pancreatic cancer [34,42]. L1CAM is considered to be a target of β-catenin, the key mediator of canonical Wnt signaling with a role in the contextual regulation of proliferation, decision points between ‘stemness’ and differentiation, cellular metabolism and EMT [39,43]. Since the microarray analysis revealed an upregulation of components that may contribute to canonical Wnt signaling in the chemoresistant cells *(Fig 3A)*, the levels of β-catenin in the chemosensitive and chemoresistant cell lines were investigated. RNA levels of β-catenin were significantly increased (P < 0.05) in the chemoresistant clones *(Fig 5A)*, but in the microarray the increase was less than two-fold and thus did not appear on the list of genes that were more than two-fold upregulated in the microarray *(S1 Table)*. Western blots show no difference in the levels of total β-catenin protein between the chemosensitive and the chemoresistant cells, neither in the cytoplasm nor in the nucleus. *(Fig 5A)*. Active β-catenin levels, as measured by an antibody detecting only active (unphosphorylated) protein, were not changed either (results not shown).

Slug did not show alterations in the microarray, which was also confirmed by RT-PCR *(Fig 5B)*. However, a Western blot analysis showed a clear upregulation of Slug protein levels in both chemoresistant cell lines compared to the chemosensitive cell lines *(Fig 5B).* To test the impact of β-catenin and Slug on the expression of L1CAM, both β-catenin and Slug were targeted by esiRNA. Knockdown of Slug, but not β-catenin, lead to a decrease in L1CAM protein levels *(Fig 5C and 5D).* When Slug and β-catenin were knocked down simultaneously in the chemoresistant cells, no further reduction in the levels of L1CAM was observed (results not shown).

When assayed for invasiveness after Slug knockdown, B1Q and B1V cells displayed reduced invasiveness compared to the esiRNA control cells (24 h invasion; *Fig 5E),* in a similar manner to what was seen with knockdown of L1CAM *(Fig 4B).* Pretreatment with L1CAM antibody in addition to Slug knockdown did not further reduce the invasive ability of the chemoresistant lines *(Fig 5E).* Although the acquisition of 5-FU-resistance induced a multitude of changes in the Panc 03.27 pancreas adenocarcinoma cells, we identified L1CAM and its regulation through Slug as a major mediator of an invasive phenotype and acquired chemoresistance, and as a potential therapeutic target.

## Discussion

5-FU was the systemic therapy of choice for advanced pancreatic cancer for many years, but in the late 90’s it was shown that the drug gemcitabine was superior to 5-FU in controlling disease-related symptoms [44]. However, 5-FU is still a recommended treatment option for pancreatic cancer today [45], and several recent studies have demonstrated a valuable role for 5-FU in combined treatment protocols compared with single gemcitabine chemotherapy [46,47].

Resistance to chemotherapeutic drugs is a major cause of treatment failure and poor prognosis in pancreatic cancer. In this study we have shown that the pancreatic cancer cell line Panc 03.27 subjected to long-term exposure to 5-FU developed resistance and acquired prototypical molecular features including upregulation of thymidylate synthase, survivin, and drug pumps. Further, we demonstrated that the chemoresistant cells became more mesenchymal-like and underwent EMT-related changes, including loss of epithelial markers (E-cadherin and CK19) and enhanced expression of the mesenchymal markers vimentin and N-cadherin, and the transcriptional factor Slug. We showed an upregulation of L1CAM in the 5-FU-resistant cells, which is in line with previous findings that support the hypothesis that L1CAM is upregulated by cancer cells as a part of the EMT process [13–18]. We were further able to link L1CAM to the process of EMT by demonstrating that the increased invasiveness of the chemoresistant cells was dependent on L1CAM, and that expression of L1CAM was dependent on Slug. In line with this observation, Gavert *et al*. showed that stimulation of pancreatic ductal epithelial cells with TGF-β1 led to the acquisition of a spindle-shaped cell morphology and upregulation of mesenchymal proteins and L1CAM expression, which was dependent on JNK-mediated activation of Slug [40].

In the breast carcinoma cell line MCF7, upregulation of L1CAM has been shown to lead to disruption of E-cadherin-containing adherens junctions and thereby to increased transcriptional activity of β-catenin [48]. Activation of β-catenin contributed to sustained L1CAM expression and enhanced cell motility [48], which is in line with other evidence showing that L1CAM is a target of β-catenin signaling [39]. However, our results did not show altered protein expression or localization of β-catenin in the chemoresistant cells, and more importantly, knockdown of β-catenin did not reduce levels of L1CAM, indicating that L1CAM expression was not regulated by β-catenin in these cells. Our results are thus in line with findings by Pfeifer *et al*. showing in endometrial carcinoma that Slug, and not β-catenin, is responsible for upregulation of L1CAM [41].

Overexpression of L1CAM has been shown to promote tumor cell proliferation, and inhibition of L1CAM expression or function can suppress proliferation [35–37]. Knockdown of L1CAM reduced proliferation in the chemoresistant Panc 03.27 cell lines *(Fig 4D)*. This reduction in growth was not seen in the chemosensitive cell lines not expressing L1CAM. Our results point towards a specific role for L1CAM in the proliferation of 5-FU-resistant pancreatic cancer cells. Further work is needed to understand the effect of L1CAM on proliferation, however, other reports point towards a link to the Erk1/2 and the Akt-pathways, which both are known to accelerate proliferation and growth of tumor cells [35,37,49,50]. Furthermore, the reduced proliferation seen for the chemoresistant clones following L1CAM knockdown in Fig 4D can partly be a result of the slight increase in apoptosis seen in Fig 4F, following L1CAM knockdown.

Interestingly, the increased invasiveness displayed by our chemoresistant clones was reduced when L1CAM was knocked down, or when the extracellular part of L1CAM was blocked with an antibody. Increased invasiveness through upregulation of L1CAM is possibly one of many regulations downstream of Slug following induction of EMT.

There are some contradictory reports on the role L1CAM plays in the process of EMT. For instance, Gavert *et al*. recently showed that L1CAM mediated metastasis of colon cancer cells was dispensable for EMT induction and an altered expression of epithelial and mesenchymal marker proteins [51]. Further studies are required to elaborate whether upregulation of L1CAM is part of the EMT or even the inducing event. L1CAM might not be an EMT-mediator itself, but appears to be regulated by EMT-induced Slug, and once expressed it seems to drive invasion.

L1CAM has been shown to be involved in the mediation of chemoresistance against gemcitabine and etoposide in PDAC cell lines [38]. We investigated whether knockdown of L1CAM reduced the acquired resistance to 5-FU displayed by the cell line B1V. In our hands, L1CAM appeared to moderately protect cells from apoptosis in the absence of 5-FU, and more so in the presence of 5-FU.

Further studies are planned to investigate whether treatment with anti-L1CAM antibodies will reduce growth or metastasis of our 5-FU-resistant pancreatic cancer cell lines *in vivo*. The finding that pancreatic cancer cells with acquired resistance to 5-FU show increased expression of L1CAM, in addition to the distinction of L1CAM presence in cancerous vs. normal tissues [52], makes us hopeful that targeting of L1CAM with therapeutic antibodies and/or in combination with 5-FU could potentially benefit selected patients with refractory pancreatic tumors. Our findings provide further insight into the molecular mechanisms leading to a chemoresistant and migratory phenotype in pancreatic cancer cells and highlight the importance of addressing Slug-induced L1CAM expression in recurrent pancreatic cancer.

## Materials and Methods

### Cells and culture conditions

Panc 03.27 pancreatic adenocarcinoma cells were obtained from ATCC (CRL-2549) and cultured in RPMI medium (R8758, Sigma-Aldrich) containing 10% FBS (16000–044, Invitrogen) and penicillin/streptomycin (P/S, 17-603E, BioWhittaker), and 500 μl ITS (I3146, Sigma-Aldrich) at 37°C in a humidified atmosphere of 5% CO_2_. Cell lines were detached using Accutase (L11–007, PAA).

### 5-FU-resistant cell line creation and clone isolation

Panc 03.27 5-FU-resistant cell lines were generated by continuous exposure of the tumor cells to 5-Fluorouracil (5-FU, F6627, Sigma-Aldrich) over a 6 month period. 5-FU was dissolved in DMSO (D8418, Sigma-Aldrich). Incubation began with 0.5 μg/ml 5-FU and increased to 1 μg/ml over time. After stable, proliferating lines resistant to 1 μg/ml 5-FU were created, clones were selected through limited dilution and clones derived from single cells were isolated and expanded over the next 3–4 months.

### 5-FU dose curve (proliferation assay)

Cells were plated at 1,500 cells per well in 96 well plates (Nunc) and allowed to attach overnight. The following morning media was replaced with 200 μl 5-FU containing media in a range from 0–10 μg/ml. The viability of the cells was determined by performing MTS assays according to manufacturer’s protocol (G3582, CellTiter 96 AQ_ueous_ Non-Radioactive Cell Proliferation Assay (MTS), Promega), and absorbance at 490 nm was measured using an SLT SPECTRA plate reader (Perkin Elmer, Waltham, Massachusetts, USA). One plate was read before addition of 5-FU to serve as the initial absorbance reading. Plates were then read daily (once every 24 hours) for 6 days and growth was calculated by subtracting the background absorbance. Data represent means n = 6 for each data point.

### Incucyte growth curves

24 hours after transfection with esiRNA, cells were detached using Accutase (L11–007, PAA) and replated at 1,500 cells per well in 96-well plates (Nunc). The plates were imaged using an IncuCyte FLR 20x (Essen BioScience, Ann Arbor, Michigan, USA), with phase contrast photos taken every 4 hours for the duration of the experiment, and % confluence was given as output. Graph in *Fig 4D* shows average percentage well coverage every 12 hours. Representative phase contrast images from the IncuCyte were used in *Fig 2A* (20x objective; scale bar attached to every image). Data represent means n = 6 for each data point.

### Microarray hybridization and analysis

Microarray hybridizations (4 replicates per condition) were performed in the Norwegian Microarray and Sequencing Core Facility (Norwegian Microarray Consortium) at the Oslo University Hospital HF. First, 500 ng of total RNA was amplified and labeled using Illumina RNA TotalPrep Amplification kit according to manufacturer’s instructions. 750 ng of biotin labeled cRNA was hybridized to Illumina’s Human HT-12 v4 Expression BeadChip according to the manufacturer's instructions. The probes on the Illumina chip are based on the content from the National Center for Biotechnology Information RefSeq database (HumanHT-12_V4_0_R2_15002873_B). The data were quantile normalized, and average of probe set signal per gene was used to calculated averages of signals across arrays in replicates. Hybridizations and differential expression was assessed by calculating fold change in the B1V line versus the Nt line.

For ontology classifications, genes upregulated at least 2 fold in B1V versus the Nt cells were fed into HOMER (Hypergeometric Optimization of Motif EnRichment) [53] sub scripts findGO to retrieve enrichment calculated assuming cumulative hypergeometric distribution. The gene ontology of biological processes according to Wiki pathways was used to determine enriched pathways and only pathways with P < 0.05 were considered. (Microarray data can be found in GEOarchive, accession number GSE58386.)

### RT-PCR

Total RNA was isolated using the GeneElute miniprep kit (Sigma) following the manufacturer's instructions. cDNA was synthesized using the SuperScript VILO kit (11754050, Life Technologies), and real-time PCR was carried out using TaqMan gene expression master mix (4369016, Life Technologies) according to the manufacturer's instructions on a StepOnePlus cycler (Life Technologies, Waltham, Massachusetts, USA). GAPDH was used to normalize the amount of cDNA in each sample and to guarantee the comparability of the calculated mRNA expression in all samples analyzed. In all real-time PCR graphs in Fig 2, the sample Nt is set to 1, and all samples are set relative to Nt. Error bars represent standard deviation. Statistically significant difference between Nt and B1V (P<0.05) is indicated by *. To assess whether the real-time PCR results were significantly different we used the two-sided Students *t*-test by Sigma plot 2001. Data represent Means ± SD; n≥3.

Probes were ordered from Life Technologies and were as follows;

ABCB1-Hs00184500_m1
ABCC5-Hs00981087_m1
CTNNB1-Hs00355049_m1
GAPDH-Hs02758991_g1
Vimentin-Hs00185584_m1
CDH1/Ecadherin-Hs01023894_m1
CDH2/Ncadherin- Hs00983056_m1
L1CAM- Hs01109748_m1
SNAI2- Hs00950344_m1

### Invasion assay

BD BioCoat matrigel invasion chamber 24-well plates; growth factor reduced; 8.0 μm pore size (354483, BD Biosciences) were used in the experiment as such; 25,000 cells (Fig 2J); 20,000 cells (Fig 4B and 4C); or 15 000 cells (Fig 5E) were plated in the top chamber in 500 μl of media containing 1% FBS and P/S. The bottom wells contained 750 μl of RPMI with 20% FBS. The cells were allowed to invade for 24 hours (Figs 2J, 4B, and 5E) or 48 (Fig 4C) hours, then the non-invading cells were scraped from the upper surface of the membrane with a cotton swab, and the cells on the lower side were stained with 0.02% crystal violet (HT90132, Sigma-Aldrich) in formaldehyde (F8775, Sigma-Aldrich). The number of invading cells was counted through the entire surface area (about 9 microscopic fields at 10x). In the antibody blocking experiment, cells were incubated in RPMI medium containing 1% FBS and P/S and either 2 μg/ml mouse anti-L1CAM antibody (UJ127.11, Sigma), or 2 μg/ml mouse IgG isotype control antibody (sc-2025, Santa Cruz) for 1 hour at 4°C, before added to invasion assay. Antibody was also present during the invasion assay. Data represent Means ±SD; n = 3 for each data point. Each assay was run at least twice.

### Western blot

Total cell extracts were made by adding cold TM buffer containing protease inhibitors (Total protein extraction kit, 2140, Millipore) to 80% confluent T25 flasks (136196, Nunc). Flasks were incubated on ice for 30 minutes and cells were collected using cell scrapers from Starstedt into 1.5 ml Eppendorf tubes, followed by centrifugation for 15 minutes at 13,000 rpm. Supernatant was transferred to another tube and protein concentration was measured using Bradford Assay (500–0205, Bio-Rad). Cytoplasmic and nuclear extracts: cells were detached using Accutase (L11–007, PAA), washed 1x in RPMI and 2x in PBS, and isolated using the Thermo NE-PER Nuclear and Cytoplasmic Extraction kit (78833, Thermo Scientific) per manufacturer’s instructions. Protein concentration was measured using Bradford assay. Protein samples were loaded on to gels (Novex Bis-Tris or Tris-Acetate gels, Life Technologies) with PageRuler prestained protein ladder (26616, Fermentas) and run in Novex electrophoresis chambers (Life technologies). Proteins were transferred to nitrocellulose membranes (LC2000, Novex, Life Technologies), blocked with 5% milk (A0830,0500, AppliChem), 0.05% tween-20 in TBS (09-7510-100, Medicago) for 1 hour, and stained with primary (4°C overnight with rocking in 5% milk, 0.05% tween-20 in TBS) and secondary antibodies (1 hour at room temperature with rocking in 5% milk, 0.1% tween-20 in TBS). Primary antibodies used; monoclonal mouse anti-active β-catenin (1:2000, 05–665, Millipore), monoclonal rabbit anti-Actin (1:2000, A5441, Sigma-Aldrich), monoclonal mouse anti-β-catenin (1:5000, 610154, BD Transduction Laboratories), polyclonal rabbit anti-CK19 (1 μg/ml, ab15463, Abcam), polyclonal rabbit anti-Lamin B1 (0.1 μg/ml, ab16048, Abcam), monoclonal rabbit anti-TS (1:1000, ab108995, Abcam), polyclonal goat anti-SLUG (1:100, sc-10436, Santa Cruz), monoclonal mouse anti-L1CAM (1:1000, L4543 (UJ127.11), Sigma-Aldrich), polyclonal rabbit anti-E-cadherin (1:500, ab15148, Abcam), monoclonal mouse anti-vimentin (1:1000, ab8978, Abcam), and polyclonal rabbit anti-survivin (1:1000, ab24479, Abcam). Secondary antibodies used are goat anti-mouse HRP or donkey anti-rabbit HRP (both 1:10000, sc-2005 and sc-2313, Santa Cruz). Bands were visualized using ECL plus (RPN2236, Amersham) on Amersham Hyperfilm (28906836, Amersham). Image Studio Lite (version 3.1.4) was used to quantify bands (normalized against loading controls).

### Flow cytometric analysis of L1CAM expression

To investigate cell surface expression of L1CAM on the Panc 03.27 cell lines, flow cytometric analysis with PE-conjugated monoclonal anti-L1CAM antibody was performed (1:100, ab95694, Abcam). Cell lines were detached using Accutase (L11–007, PAA), washed 1x in RPMI and 2x in PBS, before they were resuspended in PBS containing anti-L1CAM-PE antibody, diluted 1:100. Cells were incubated at room temperature for 1 hour, before they were washed twice in PBS after antibody incubation. Acquisition of data was performed using an EasyCyte flow cytometer (GuavaTechnologies). A minimum of 5,000 cells were acquired per sample and exclusion of non-viable cells and debris was based on lower forward scatter and side scatter properties. Data analysis was performed using Flowing Software (Version 2.5.1).

### Annexin V Assay (Flow cytometry)

To perform Annexin V assay on the Panc 03.27 cell lines, Annexin V FITC Assay (BD Biosciences) was used. Following esiRNA transfection and/or 5-FU treatment, cell lines were detached using Accutase, and spun at 300 rpm for 5 minutes together with used medium to collect released/dead cells. Medium was removed, and Annexin V was added to cells according to product protocol. Acquisition of data was performed using an EasyCyte flow cytometer (GuavaTechnologies). Exclusion of non-viable cells and debris was based on lower forward scatter and side scatter properties. Data analysis was performed using Flowing Software (Version 2.5.1).

### Immunofluorescence

Cells were plated at 20,000 cells/well on coverslips in 24 well plates and allowed to attach overnight. Wells were fixed, permeabilized, and blocked as described previously [54]. Cells stained on cover slips were mounted on slides using Fluorescence Mounting Medium (S3023, DAKO). The coverslips were then incubated for 16 hours at 4°C with polyclonal rabbit anti-CK19 (10 μg/ml, ab15463, Abcam) or polyclonal rabbit anti-N-cadherin (1:100, ab76057, Abcam). Alexa Fluor 594 polyclonal goat anti-rabbit (1:700, A-11012, Life Technologies) was used as a secondary antibody in 1% BSA (A2153, Sigma-Aldrich) in PBS for 1 hour at room temperature. For L1CAM, monoclonal anti-L1CAM-PE (1:50, ab95694, Abcam) was used. Nuclei were counterstained with DAPI (1 μg/ml, 10236276001, Roche,), in PBS for 5 minutes at room temperature. Images were obtained using an Axiovert 200M fluorescence microscope (Zeiss Axiovert 200 M, Carl Zeiss MicroImaging GmbH, Jena, Germany) with CCD camera (Zeiss Axiocam HR), using Axiovision software. All images are taken with 40x magnification.

### esiRNA knockdown of β-catenin, L1CAM, and Slug

300,000 cells per well were plated in 6-well plates and allowed to attach overnight in an incubator at 37°C, and with 5% CO_2_. The following morning, 30 nM of MISSION esiRNA targeting human CTNNB1 (β-catenin, EHU139421), human L1CAM (EHU100991), or human SNAI2 (Slug, EHU048191) or MISSION esiRNA EGFP (EHUEGFP) as ctrl (all from Sigma) per well was transfected using Lipofectamine 2000 (11668–019, Life Technologies), as per instructions. Cells were then returned to the incubator for 24 hours. After 24 hours, the cells were either detached and plated for further experiments (Immunostain, matrigel invasion assay) or left for 72 hours after change of medium (for RNA- and protein extractions).

### Statistical analysis

To assess whether the data was significantly different we used the two-sided Student’s t- test by Sigma plot 2001. A minimum significance level of P < 0.05 was used.

## Supporting Information

S1 Fig Cluster analysis of gene expression in B1V and Nt. 607 genes with log2 change >1 were considered.
(PDF)

S1 Table Upregulated genes. List of 319 genes upregulated at least 2-fold in the B1V clone compared to Nt clone of the Panc 03.27 cell line (P <0.05).
(PDF)

S2 Table Downregulated genes. List of 288 genes downregulated at least 2-fold in the B1V clone compared to Nt clone of the Panc 03.27 cell line (P <0.05).
(PDF)
