# Supplementary material for: Slug-Dependent Upregulation of L1CAM Is Responsible for the Increased Invasion Potential of Pancreatic Cancer Cells following Long-Term 5-FU Treatment
Source: PLoS One. 2015 Apr 10;10(4):e0123684. doi: 10.1371/journal.pone.0123684 (PMC4393253; doi:10.1371/journal.pone.0123684)
Supplement: S1 Fig — 607 genes with log2 change >1 were considered. (PDF) [file pone.0123684.s001.pdf]

Supplementary Figure S1. Cluster analysis of gene expression in B1V and Nt. 607 genes with log2 change >1 were considered.

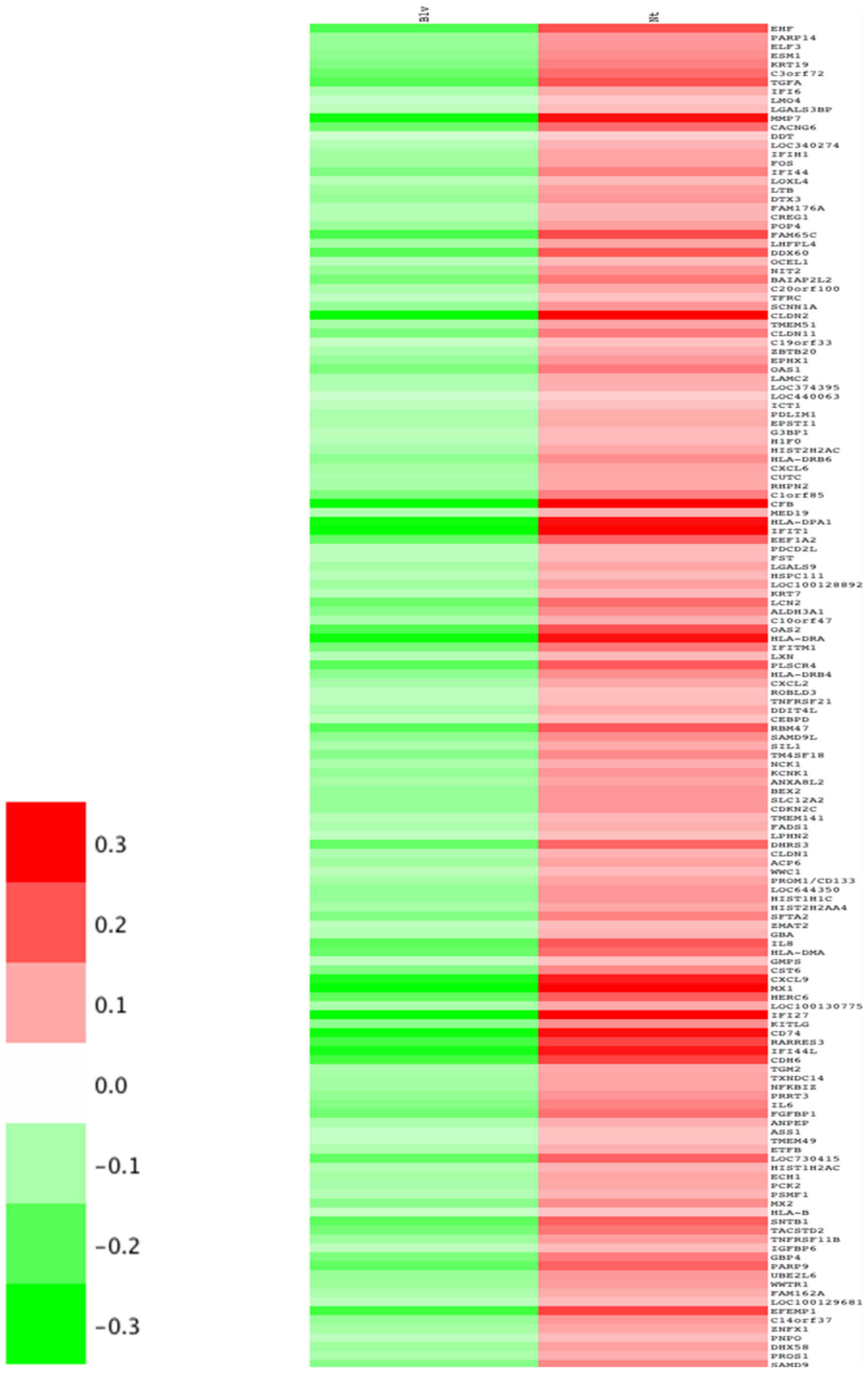

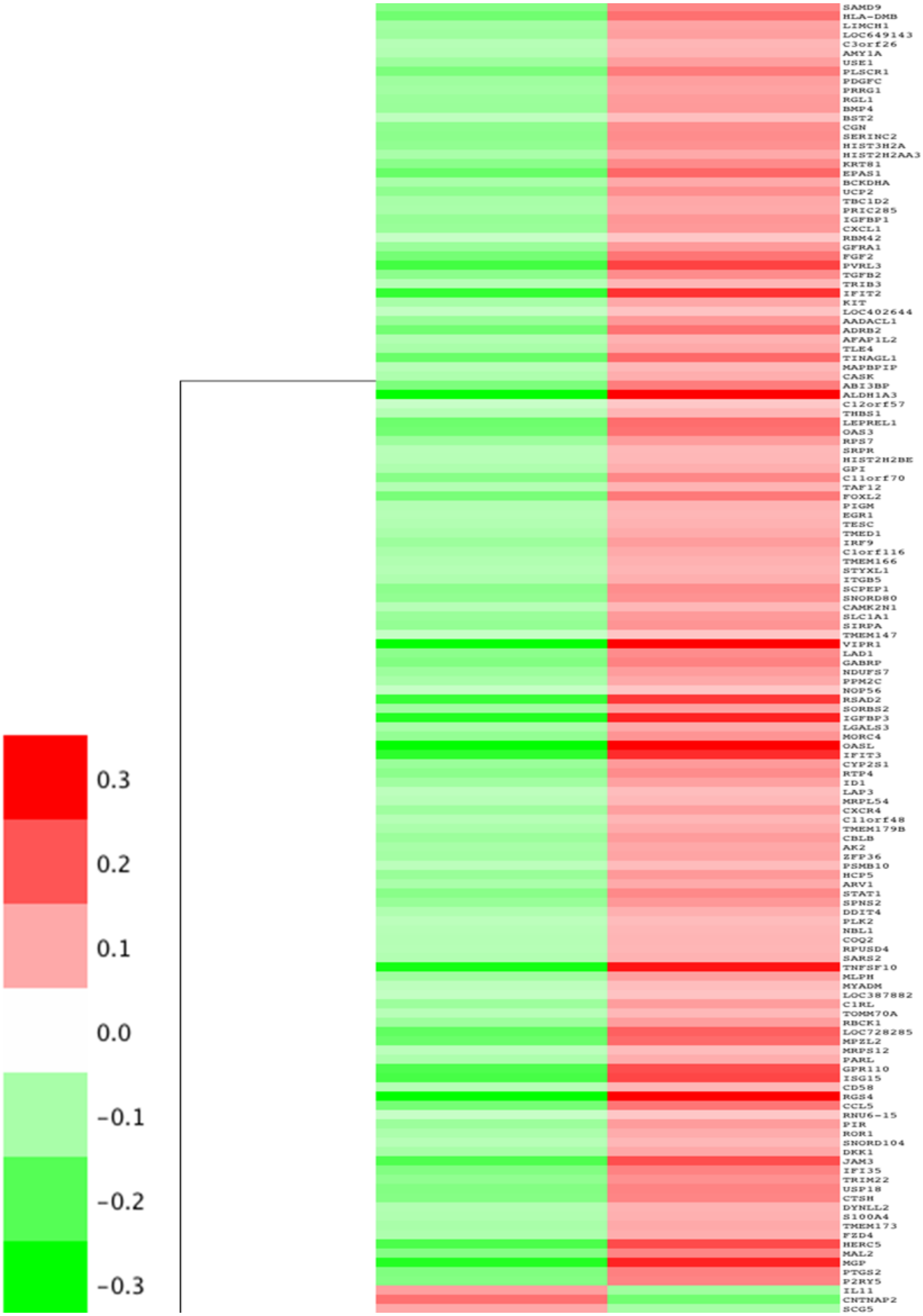

SAMD9
HLA-DMB
LIMCH1
LOC649143
C3orf26
AMY1A
USE1
PLSCR1
PDGFC
PRRG1
RGL1
BMP4
BST2
CGN
SERINC2
HIST3H2A
HIST2H2AA3
KRT81
EPAS1
BCKDHA
UCP2
TBC1D2
PRIC285
IGFBP1
CXCL1
RBM42
GFRA1
FGF2
PVRL3
TGFB2
TRIB3
IFIT2
KIT
LOC402644
AADACL1
ADRB2
AFAP1L2
TLE4
TINAGL1
MAPBPIP
CASK
ABI3BP
ALDH1A3
C12orf57
THBS1
LEPREL1
OAS3
RPS7
SRPR
HIST2H2BE
GPI
C11orf70
TAF12
FOXL2
PIGM
EGR1
TESC
TMED1
IRF9
C1orf116
TMEM166
STYXL1
ITGB5
SCPEP1
SNORD80
CAMK2N1
SLC1A1
SIRPA
TMEM147
VIPR1
LAD1
GABRP
NDUFS7
PPM2C
NOP56
RSAD2
SORBS2
IGFBP3
LGALS3
MORC4
OASL
IFIT3
CYP2S1
RTP4
ID1
LAP3
MRPL54
CXCR4
C11orf48
TMEM179B
CBLB
AK2
ZFP36
PSMB10
HCP5
ARV1
STAT1
SPNS2
DDIT4
PLK2
NBL1
COQ2
RPUSD4
SARS2
TNFSF10
MLPH
MYADM
LOC387882
C1RL
TOMM70A
RBCK1
LOC728285
MPZL2
MRPS12
PARL
GPR110
ISG15
CD58
RGS4
CCL5
RNU6-15
PIR
ROR1
SNORD104
DKK1
JAM3
IFI35
TRIM22
USP18
CTSH
DYNLL2
S100A4
TMEM173
FZD4
HERC5
MAL2
MGP
PTGS2
P2RY5
IL11
CNTNAP2
SCG5
0.3
0.2
0.1
0.0
-0.1
-0.2
-0.3

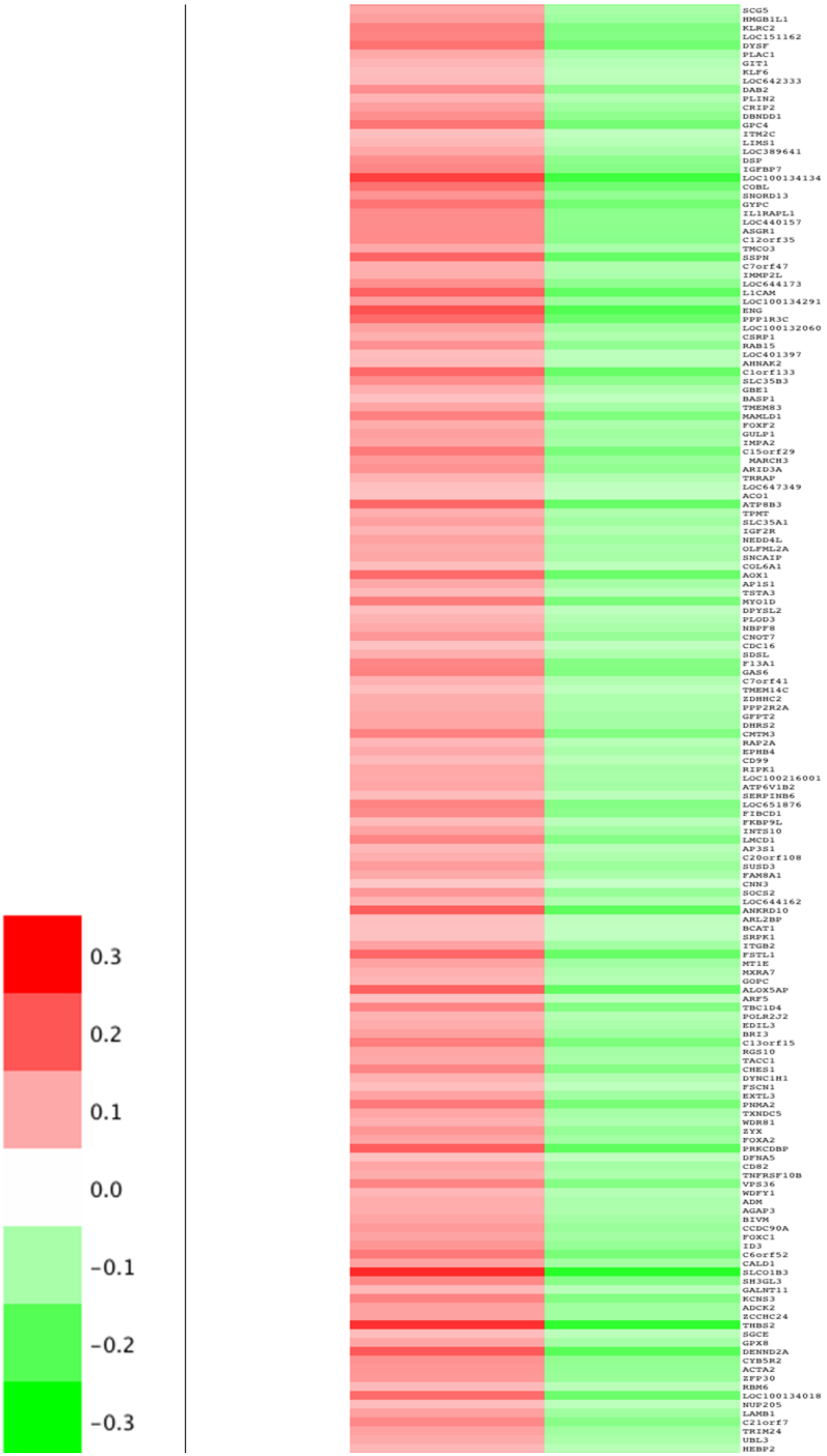
0.3
0.2
0.1
0.0
-0.1
-0.2
-0.3
SCG5
HMGB1L1
KLRC2
LOC151162
DYSF
PLAC1
GIT1
KLF6
LOC642333
DAB2
PLIN2
CRIP2
DBNDD1
GPC4
ITM2C
LIMS1
LOC389641
DSP
IGFBP7
LOC100134134
COBL
SNORD13
GYPC
IL1RAPL1
LOC440157
ASGR1
C12orf35
TMCO3
SSPN
C7orf47
IMMP2L
LOC644173
L1CAM
LOC100134291
ENG
PPP1R3C
LOC100132060
CSRP1
RAB15
LOC401397
AHNAK2
C1orf133
SLC35B3
GBE1
BASP1
TMEM83
MAMLD1
FOXF2
GULP1
IMPA2
C15orf29
MARCH3
ARID3A
TRRAP
LOC647349
ACO1
ATP8B3
TPMT
SLC35A1
IGF2R
NEDD4L
OLFML2A
SNCAIP
COL6A1
AOX1
AP1S1
TSTA3
MYO1D
DPYSL2
PLOD3
NBPF8
CNOT7
CDC16
SDSL
F13A1
GAS6
C7orf41
TMEM14C
ZDHHC2
PPP2R2A
GFPT2
DHRS2
CMTM3
RAP2A
EPHB4
CD99
RIPK1
LOC100216001
ATP6V1B2
SERPINB6
LOC651876
FIBCD1
FKBP9L
INTS10
LMCD1
AP3S1
C20orf108
SUSD3
FAM8A1
CNN3
SOCS2
LOC644162
ANKRD10
ARL2BP
BCAT1
SRPK1
ITGB2
FSTL1
MT1E
MXRA7
GOPC
ALOX5AP
ARF5
TBC1D4
POLR2J2
EDIL3
BRI3
C13orf15
RGS10
TACC1
CHES1
DYNC1H1
FSCN1
EXTL3
PNMA2
TXNDC5
WDR81
ZYX
FOXA2
PRKCDBP
DFNA5
CD82
TNFRSF10B
VPS36
WDFY1
ADM
AGAP3
BIVM
CCDC90A
FOXC1
ID3
C6orf52
CALD1
SLCO1B3
SH3GL3
GALNT11
KCNS3
ADCK2
ZCCHC24
THBS2
SGCE
GPX8
DENND2A
CYB5R2
ACTA2
ZFP30
RBM6
LOC100134018
NUP205
LAMB1
C21orf7
TRIM24
UBL3
HEBP2

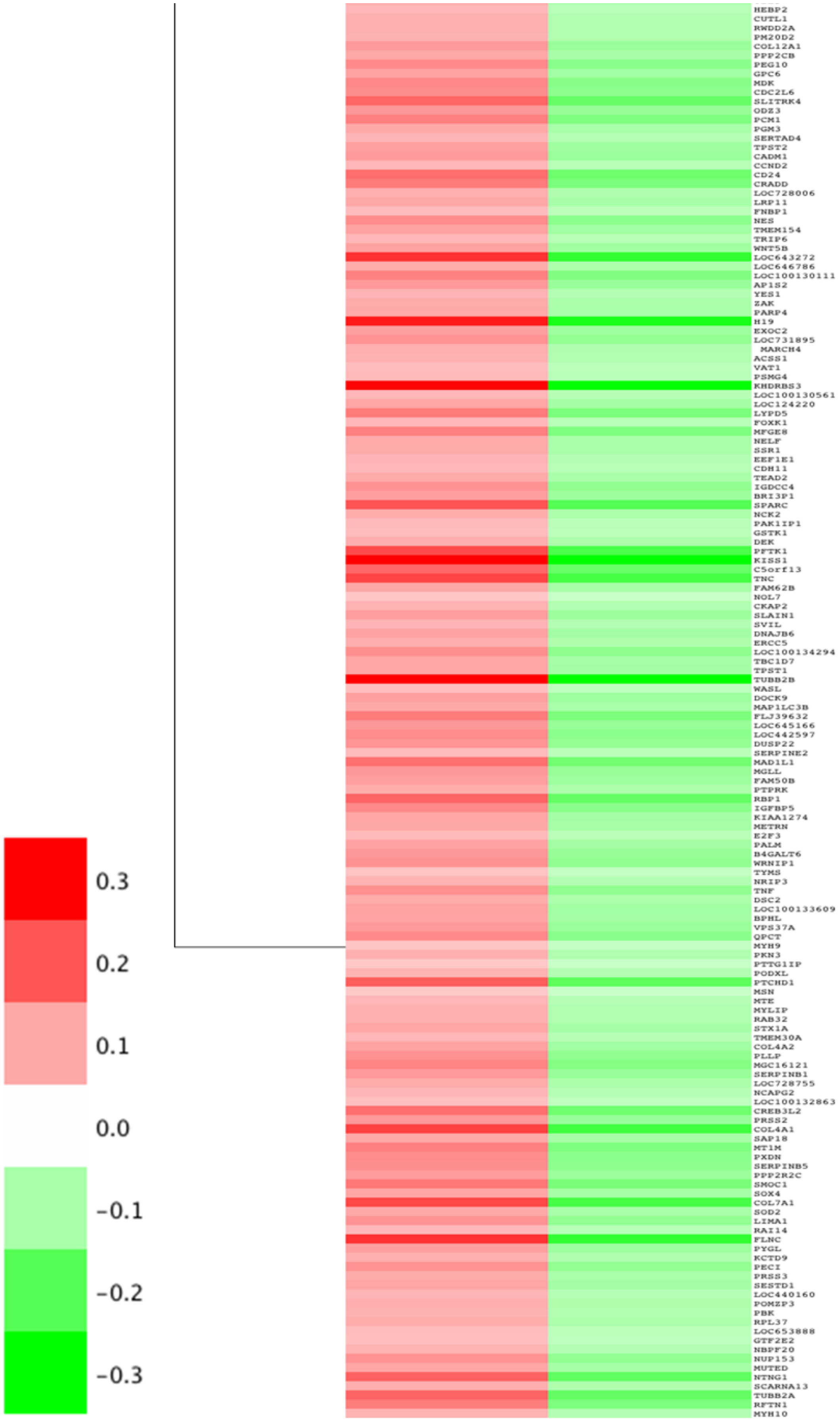
HEBP2
CUTL1
RWDD2A
PM20D2
COL12A1
PPP2CB
PEG10
GPC6
MDK
CDC2L6
SLITRK4
ODZ3
PCM1
PGM3
SERTAD4
TPST2
CADM1
CCND2
CD24
CRADD
LOC728006
LRP11
FNBP1
NES
TMEM154
TRIP6
WNT5B
LOC643272
LOC646786
LOC100130111
AP1S2
YES1
ZAK
PARP4
H19
EXOC2
LOC731895
MARCH4
ACSS1
VAT1
PSMG4
KHDRBS3
LOC100130561
LOC124220
LYPD5
FOXK1
MFGE8
NELF
SSR1
EEF1E1
CDH11
TEAD2
IGDCC4
BRI3P1
SPARC
NCK2
PAK1IP1
GSTK1
DEK
PFTK1
KISS1
C5orf13
TNC
FAM62B
NOL7
CKAP2
SLAIN1
SVIL
DNAJB6
ERCC5
LOC100134294
TBC1D7
TPST1
TUBB2B
WASL
DOCK9
MAP1LC3B
FLJ39632
LOC645166
LOC442597
DUSP22
SERPINE2
MAD1L1
MGLL
FAM50B
PTPRK
RBP1
IGFBP5
KIAA1274
METRN
E2F3
PALM
B4GALT6
WRNIP1
TYMS
NRIP3
TNF
DSC2
LOC100133609
BPHL
VPS37A
QPCT
MYH9
PKN3
PTTG1IP
PODXL
PTCHD1
MSN
MTE
MYLIP
RAB32
STX1A
TMEM30A
COL4A2
PLLP
MGC16121
SERPINB1
LOC728755
NCAPG2
LOC100132863
CREB3L2
PRSS2
COL4A1
SAP18
MT1M
PXDN
SERPINB5
PPP2R2C
SMOC1
SOX4
COL7A1
SOD2
LIMA1
RAI14
FLNC
PYGL
KCTD9
PECI
PRSS3
SESTD1
LOC440160
POMZP3
PBK
RPL37
LOC653888
GTF2E2
NBPF20
NUP153
MUTED
NTNG1
SCARNA13
TUBB2A
RFTN1
MYH10
0.3
0.2
0.1
0.0
−0.1
−0.2
−0.3
