# Supplementary material for: Slug-Dependent Upregulation of L1CAM Is Responsible for the Increased Invasion Potential of Pancreatic Cancer Cells following Long-Term 5-FU Treatment
Source: PLoS One. 2015 Apr 10;10(4):e0123684. doi: 10.1371/journal.pone.0123684 (PMC4393253; doi:10.1371/journal.pone.0123684)
Supplement: S1 Table — List of 319 genes upregulated at least 2-fold in the B1V clone compared to Nt clone of the Panc 03.27 cell line (P <0.05). (PDF) [file pone.0123684.s002.pdf]

Supplementary Table S1. List of 319 genes upregulated at least 2-fold in the chemoresistant cell line B1V versus the chemosensitive cell line Nt. (P <0.05) (FC; fold change.)

List of 319 genes upregulated at least 2-fold in the chemoresistant cell line B1V versus the chemosensitive cell line Nt									
**Gene name**	**FC B1V/Nt**	**Gene name**	**FC B1V/Nt**	**Gene name**	**FC B1V/Nt**	**Gene name**	**FC B1V/Nt**	**Gene name**	**FC B1V/Nt**
PLAC1	2,00	LOC440160	2,16	HEBP2	2,35	IL1RAPL1	2,68	C5orf13	3,62
COL6A1	2,00	CNN3	2,17	MYH10	2,36	SOD2	2,68	LOC100134018	3,65
GALNT11	2,01	LOC642333	2,17	DOCK9	2,36	DSP	2,68	GPC4	3,67
FOXF2	2,01	KLF6	2,17	EEF1E1	2,36	TPST1	2,69	PNMA2	3,69
CDC16	2,01	RIPK1	2,17	TMEM14C	2,36	LIMA1	2,69	SLITRK4	3,72
ZDHHC2	2,01	LOC124220	2,17	BASP1	2,36	LOC100134294	2,69	LOC151162	3,73
MXRA7	2,02	KCTD9	2,18	TRIP6	2,37	F13A1	2,71	CHES1	3,80
PKN3	2,02	ITM2C	2,18	PODXL	2,37	BRI3P1	2,71	NTNG1	3,83
ACSS1	2,02	IMMP2L	2,18	PPP2CB	2,37	FIBCD1	2,71	MFGE8	3,86
NRIP3	2,02	LOC728006	2,18	PRSS3	2,38	SLC35A1	2,72	PTCHD1	3,89
RBM6	2,02	SNCAIP	2,18	FAM50B	2,38	QPCT	2,73	MAD1L1	3,90
SCG5	2,03	SERPINE2	2,19	PPP2R2A	2,38	SSR1	2,74	ALOX5AP	3,91
FOXK1	2,03	GULP1	2,19	STX1A	2,39	SH3GL3	2,76	GAS6	3,91
AGAP3	2,03	CDH11	2,19	ODZ3	2,39	MGLL	2,77	IGFBP7	3,91
RAP2A	2,03	GSTK1	2,19	TMCO3	2,39	DUSP22	2,79	COBL	3,94
GTF2E2	2,03	SESTD1	2,20	FOXA2	2,40	TACC1	2,80	RFTN1	3,99
GOPC	2,03	POLR2J2	2,20	DYNC1H1	2,40	PLLP	2,81	PCM1	4,06
FNBP1	2,03	LOC644162	2,20	INTS10	2,40	FAM62B	2,81	C12orf35	4,08
SCARNA13	2,03	C7orf41	2,20	SGCE	2,40	SOX4	2,81	SSPN	4,09
SRPK1	2,04	IGF2R	2,20	ID3	2,41	MT1E	2,82	PRKCDBP	4,16
ARL2BP	2,04	SAP18	2,21	EXOC2	2,42	LOC651876	2,84	L1CAM	4,24
FKBP9L	2,04	NELF	2,21	COL12A1	2,42	MT1M	2,84	PPP1R3C	4,47
SERTAD4	2,04	RAI14	2,22	SUSD3	2,42	DNAJB6	2,87	SPARC	4,58
CUTL1	2,05	PALM	2,22	MAP1LC3B	2,43	HMGB1L1	2,87	C13orf15	4,64
LIMS1	2,05	DSC2	2,22	TEAD2	2,44	DEK	2,89	C1orf133	4,75
NCAPG2	2,06	ERCC5	2,22	YES1	2,44	KCNS3	2,90	ANKRD10	4,76
PAK1IP1	2,06	CD82	2,22	TNF	2,44	NES	2,92	TUBB2A	4,77
MTE	2,06	POMZP3	2,22	BIVM	2,44	CCDC90A	2,93	DYSF	4,78
BCAT1	2,06	SLAIN1	2,23	TBC1D7	2,45	SLC35B3	2,93	CREB3L2	4,79
PLIN2	2,06	UBL3	2,23	PRSS2	2,45	LOC440157	2,97	ENG	4,93
SDSL	2,06	PM20D2	2,23	RAB32	2,46	KLRC2	3,00	PFTK1	4,93
LOC653888	2,06	NBPF8	2,23	LRP11	2,46	PYGL	3,01	CD24	5,02
MARCH4	2,07	WDFY1	2,23	ZFP30	2,47	DBNDD1	3,01	DENND2A	5,13
DFNA5	2,07	MUTED	2,23	EPHB4	2,48	CNOT7	3,01	COL4A1	5,35
PBK	2,07	TRIM24	2,23	FSCN1	2,49	MYO1D	3,03	RBP1	5,40
TSTA3	2,07	GFPT2	2,24	CALD1	2,49	TPST2	3,03	LOC100134134	5,62
AHNAK2	2,07	MYLIP	2,24	FOXC1	2,49	MAMLD1	3,05	FSTL1	5,65
PSMG4	2,07	PLOD3	2,25	IGDCC4	2,49	C6orf52	3,07	COL7A1	5,73
WDR81	2,07	TNFRSF10B	2,25	CRIP2	2,49	CDC2L6	3,09	TNC	6,15
TPMT	2,08	LOC401397	2,25	LOC731895	2,50	ZYX	3,09	FLNC	6,34
MSN	2,08	TYMS	2,25	CYB5R2	2,50	DAB2	3,10	THBS2	6,51
RPL37	2,08	MYH9	2,25	ZCCHC24	2,50	AP1S2	3,11	SLCO1B3	7,20
WASL	2,08	PPP2R2C	2,26	LOC644173	2,50	LYPD5	3,11	LOC643272	8,06
GIT1	2,08	LOC646786	2,26	CSRP1	2,50	LOC100130111	3,13	H19	8,10
LOC647349	2,08	CKAP2	2,26	CCND2	2,51	LAMB1	3,14	KHDRBS3	12,87
NOL7	2,09	GPX8	2,26	ITGB2	2,51	NUP153	3,17	TUBB2B	14,10
VAT1	2,09	TMEM30A	2,27	C20orf108	2,51	SERPINB1	3,18	KISS1	16,28
PTTG1IP	2,09	NBPF20	2,27	BPHL	2,53	TBC1D4	3,23		
TMEM83	2,09	KIAA1274	2,28	IMPA2	2,53	MGC16121	3,23		
METRN	2,09	AP3S1	2,29	CADM1	2,54	BRI3	3,23		
LOC728755	2,09	IL11	2,30	VPS37A	2,55	GYPC	3,24		
SVIL	2,10	ZAK	2,31	ATP6V1B2	2,57	PECI	3,26		
FAM8A1	2,11	MARCH3	2,31	AP1S1	2,59	C15orf29	3,27		
ARF5	2,12	LOC100130561	2,31	RGS10	2,59	FLJ39632	3,27		
NUP205	2,13	GBE1	2,32	ARID3A	2,60	LOC645166	3,29		
CD99	2,14	PGM3	2,33	B4GALT6	2,60	VPS36	3,32		
SERPINB6	2,14	C7orf47	2,33	ASGR1	2,60	C21orf7	3,39		
LOC389641	2,14	LOC100132060	2,33	SERPINB5	2,61	LOC442597	3,40		
E2F3	2,14	LOC100216001	2,33	ADM	2,62	WRNIP1	3,41		
EDIL3	2,14	EXTL3	2,34	NEDD4L	2,64	CMTM3	3,50		
LOC100133609	2,14	GPC6	2,34	ACTA2	2,64	SMOC1	3,51		
COL4A2	2,15	NCK2	2,34	TXNDC5	2,64	CNTNAP2	3,51		
RWDD2A	2,15	TMEM154	2,34	SOCS2	2,64	PEG10	3,52		
DHRS2	2,16	DPYSL2	2,34	PXDN	2,65	CRADD	3,56		
LOC100132863	2,16	LOC100134291	2,34	IGFBP5	2,65	MDK	3,58		
WNT5B	2,16	PTPRK	2,34	PARP4	2,66	LMCD1	3,58		
ACO1	2,16	OLFML2A	2,35	RAB15	2,66	ATP8B3	3,59		
TRRAP	2,16	ADCK2	2,35	SNORD13	2,67	AOX1	3,60		
